# Decisions of the FDA on premarket tobacco product applications: Changes in the number of unique devices and liquids used by US adults who frequently use electronic nicotine delivery systems, 2020–2023

**DOI:** 10.18332/tid/184240

**Published:** 2024-03-13

**Authors:** Elizabeth Crespi, Jeffrey J. Hardesty, Qinghua Nian, Joanna E. Cohen

**Affiliations:** 1 Institute for Global Tobacco Control, Department of Health, Behavior and Society, Johns Hopkins Bloomberg School of Public Health, Johns Hopkins University, Baltimore, United States

**Keywords:** tobacco, ENDS, regulatory science, policy, survey

## Abstract

**INTRODUCTION:**

The majority of decisions on electronic nicotine delivery system (ENDS) premarket tobacco product applications (PMTAs) were made from October 2020 to February 2023; 99% (>25 million) had determinations by March 2023 and just twenty-three received marketing granted orders. We examined the unique devices and liquids used among US adults frequently using ENDS before, during, and after a majority of PMTA decisions were made.

**METHODS:**

Data are from waves 1-5 (W1: May–Oct 2020, n=1179; W5: Feb–Apr 2023, n=1290) of a longitudinal survey of US adults (≥21 years) using ENDS ≥5 days/week. User-submitted photos of participants’ most used devices and liquids were coded. Descriptive analyses and Wilcoxon signed-rank tests were used to understand the number and types of unique devices and liquids used in W1-W5, and the top brands in each wave.

**RESULTS:**

From W1-W5, the number of unique ENDS device models and liquid products used by participants increased from 279 to 357 (p<0.001) and 546 to 695 (p<0.001), respectively. More unique devices in W5 versus W1 were disposable (W1: 16.5%; W5: 36.1%); fewer were disposable pod (W1: 6.5%; W5: 3.1%) or tank (W1: 53.8%; W5: 30.8%) devices. Liquids were primarily sweet-flavored (W1: 81.1%; W5: 82.0%). The median liquid nicotine concentration increased from 12 to 50 mg/mL. In W5, few participants used FDA-approved devices (n=17; 1.3%) or liquids (n=6; 0.5%), and Elf Bar was the most commonly used device and liquid brand. Results for all waves are reported.

**CONCLUSIONS:**

Despite PMTA decisions, an increase in the number of unique device models and liquid products used among adults who frequently use ENDS was observed from 2020 to 2023. Few participants in 2023 were using FDA-approved devices or liquids. Further research and monitoring are needed to inform how FDA prioritizes enforcement actions and what types of enforcement actions are effective.

## INTRODUCTION

Appropriate and effective electronic nicotine delivery system (ENDS) regulation is critical for the protection of public health^1,2^. In the US, the Food and Drug Administration (FDA) regulates tobacco and nicotine products, including ENDS. Prior to selling a new tobacco product, companies must submit and receive approval based on a premarket tobacco product application (PMTA), which provides information about the product, such as the ingredients, potential health risks and benefits, and methods used in manufacturing and processing^3^. Alternatively, ENDS manufacturers may obtain authorization through the substantial equivalence pathway, which requires evidence that the product is as safe and effective as a current legally marketed product; to date, no ENDS products have been authorized through this process^4^.

The PMTA review process comprises four phases: 1) acceptance review, 2) filing review, 3) application review and action, and 4) postmarket reporting^3^. In reviewing PMTAs, the FDA must consider the risks and benefits to public health, including potential cessation benefits among those who currently use tobacco products and risks of initiation among those who do not use any tobacco products^3^. From October 2019 to February 2023, over 26 million PMTAs for ENDS products were submitted. The majority of decisions on these ENDS PMTAs were made from October 2020 to February 2023^5^. As of March 2023, the FDA has made determinations on over 99% of these PMTAs^6^. Of the over 26 million submissions, about 6.7 million were accepted, 1.1 million were filed and only 23 (ten refillable pod devices with eleven associated liquids and two disposable devices of the same brand but different flavors) have been issued marketing granted orders (MGOs)^5^. In addition to these 23 approved products, ‘deemed products’ that were on the market on or prior to 8 August 2016 for which PMTAs were submitted by 9 September 2020, may be sold assuming FDA has not yet issued a determination on the product^7^. Products that FDA did not accept, did not file, or denied (i.e. issued a marketing denial order) are illegal to sell.

Given the substantial progress FDA has made in reviewing and issuing determinations on PMTAs, there is a need to understand if the FDA regulatory actions were followed by a change in the ENDS devices and liquids on the market, and used by adults in the US. To our knowledge, current research in this area is limited. One study using scanner data from January 2020 to December 2022 reported an increase in ENDS units sold overall, the number of device brands sold, and disposable device unit sales^8^. Using the same scanner data, another study from 2017 to 2022 found a 293.6% increase in unit sales of ENDS, a growing proportion of which were products containing at least 5% nicotine; this trend of increasing nicotine concentration was observed across all flavors^9^. A third report using different scanner data showed a 400% increase in the number of unique ENDS products on the market, from 453 to 2023, from June 2021 to 2022^10^. However, given that these studies took place before 2023, when a majority of PMTA decisions had been made, these prior studies have not examined the extent of unique liquid brands on the market or the specific characteristics of the devices and liquids before and after the majority of PMTAs were reviewed. We fill this gap by examining the quantity and characteristics of unique devices and liquids used among US adults frequently using ENDS at five timepoints occurring before, during, and after a majority of determinations had been made on ENDS PMTAs (2020–2023).

## METHODS

### Study sample and protocols

Data are from waves 1 (May–Oct 2020; n=1179), 2 (Dec 2020–Apr 2021; n=1187), 3 (Sept–Dec 2021; n=1219), 4 (Jul–Sept 2022; n=1224), and 5 (Feb–Apr 2023; n=1290) of the Vaping and Patterns of E-cigarette Use Research (VAPER) study, a longitudinal survey of US adults (aged ≥21 years) who use ENDS ≥5 days/week examining ENDS use patterns and behaviors. Participants reported on and submitted photos of their most used ENDS device and the most used liquid with that device via an online REDCap survey. Rigorous data review and cleaning procedures were employed to ensure high quality data. Additional information about study protocols, including data review, is available elsewhere^11^; of note, the VAPER sample is similar to the nationally representative sample of adults frequently using ENDS in the 2019 Tobacco Use Supplement to the Current Population Survey (TUS-CPS) in regard to gender, race, age, and region, though the VAPER sample includes a larger proportion of lower income individuals^11^.

### Measurements

*Overview of the photo coding process*

Variables utilized in these analyses were obtained from photos of participants’ most used ENDS devices and liquids. Photos submitted by participants were examined for any identifying visual elements (e.g. words, symbols) which were then searched in Google. Websites were reviewed to find an exact visual match for the product and identify the brand and model (for devices) or brand and flavor (for liquids). Once the brand and model (for devices) or brand and flavor (for liquids) had been identified, any remaining variables of interest (described below) were coded using information directly available in the photo (e.g. nicotine concentration on a bottle) or by searching the brand and model (for devices) or brand and flavor (for liquids) along with the name of the characteristic (e.g. ‘Elf Bar BC5000 Blue Razz Ice nicotine concentration’). Manufacturer, academic, retail, and review (in this order of prioritization) sources were then reviewed to identify each characteristic. All coders were trained and assessed via several practice rounds of coding to ensure at least 90% reliability across coders. Utilizing a standard operating procedure detailing how to record the information, a single coder then searched for and coded information for each device and liquid into a pre-existing shared spreadsheet.

*Device variables*

Device variables were coded using the process described above and included device brand, model, and type (disposable device, reusable device with disposable pods, reusable device with refillable pods, reusable tank device).

*Liquid variables*

Liquid variables included in these analyses were coded using the process described above and included liquid brand, flavor, nicotine concentration (mg/mL), formulation (salt, freebase), PG/VG (propylene glycol/vegetable glycerin) ratio (PG ≤30%/VG ≥70%; PG >30% and <50%/VG >50% and <70%; PG ≥50%/VG ≤50%), container type (disposable device, disposable pod, liquid bottle), and flavor category (tobacco, menthol/mint, sweet, other). To determine the liquid flavor category, the flavor descriptions in photos or on websites were reviewed and coded for the primary flavor category (e.g. dessert, fruit, menthol/mint, tobacco) and subcategory (e.g. custard, strawberry) based on the ENDS liquid flavor wheel developed by Krüsemann et al.^12^. These categories were further collapsed into tobacco, menthol/mint, sweet, and other. Sweet included liquids with a primary flavor category of dessert, candy, fruit, or other sweets (e.g. chocolate, vanilla). Liquids with a primary flavor category of other beverages or alcohol were also considered sweet if the primary flavor subcategory was sweet (e.g. orange juice, pina colada). Note that, because the liquids for disposable devices are contained within the device itself and not sold separately, the liquid brand is the same as the device brand.

We then combined the liquid brand, flavor, nicotine concentration, formulation, and PG/VG ratio to create a variable representing the unique ENDS liquid product. We elected to include nicotine concentration, formulation, and PG/VG in the liquid product variable given their relevance in ENDS product appeal, aerosol formation, aerosol nicotine level, and/or toxicity (e.g. Juul Menthol 59 mg/mL salt liquid with 30/70 PG/VG would be one unique product and Juul Menthol 35 mg/mL salt liquid with 30/70 PG/VG would be another unique product)^13^.

### Statistical analysis

If the device or liquid photos submitted by a participant were insufficient for identifying the device brand and model or the liquid brand, flavor, nicotine concentration, formulation, and PG/VG the record was excluded from these analyses. Descriptive statistics were utilized to describe the number of unique device brands and models and liquid brands and products identified in this study, as well as the characteristics of these unique models and products and the top five most commonly used device and liquid brands. Wilcoxon signed-rank tests were utilized to test the changes in the number of unique device models and liquid products across waves. Data from each wave were analyzed cross-sectionally using STATA V.16.1.

### Ethical considerations

The Virginia Commonwealth University (No. HM20015004) and Johns Hopkins Bloomberg School of Public Health Institutional Review Boards (No. 9277) approved all study protocols. Participants provided informed consent. Study data utilized here were de-identified.

## RESULTS

### ENDS devices

**Figure 1:**
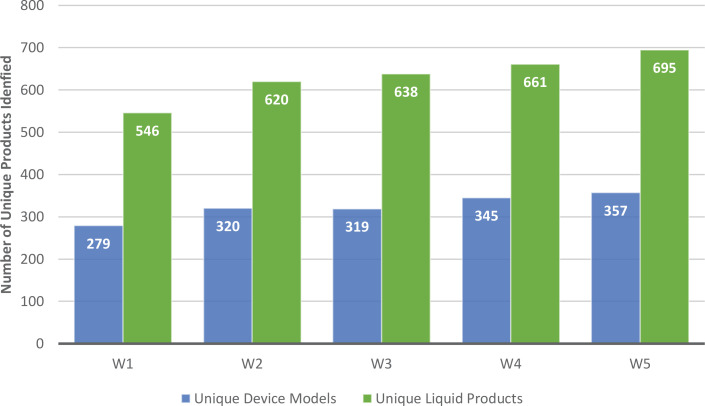
Number of unique device models and liquid products across five waves of the Vaping and Patterns of E-cigarette Use Research study, an online longitudinal survey of adults who frequently use ENDS (W1 vs W5; p<0.05)

Participants’ device brand and model could be identified for: 94.9% (n=1119) of participants in W1; 96.9% (n=1150) in W2; 97.6% (n=1990) in W3; 97.8% (n=1197) in W4; and 97.8% (n=1261) in W5. Across W1-5, the number of unique device models increased from 279 in W1 to 357 in W5 (p<0.001) (Figure 1). Accordingly, the number of unique device brands increased across waves from 92 in W1 to 121 in W5; the average number of models identified per brand did not change substantially across waves (Table 1). Note that the number of participants increased across waves (W1: n=1179; W5: n=1290); however, when we standardize the number of unique models by dividing by the sample size (number of models/n), the number of unique device models identified per person also increased (W1: 279/1179=0.24; W5: 357/1290=0.28). While most unique device models in W1 were refillable tank devices (53.8%), most device models in W5 were disposable devices (36.1%) (Table 2).

**Table 1:** Number of unique device brands and models and liquid brands and products across five waves of the Vaping and Patterns of E-cigarette Use Research study, an online longitudinal survey of adults who frequently use ENDS

	*W1*	*W2*	*W3*	*W4*	*W5*
**Unique devices**					
Brands	92	109	116	119	121
Models^a^	279	320	319	345	357
Number of models per brand	3.0	2.9	2.8	2.9	3.0
**Unique liquids**					
Brands	243	246	249	204	206
Products^b^	546	620	638	661	695
Number of products per brand	2.2	2.5	2.6	3.2	3.4

a Wilcoxon signed rank test p-values for the prevalence of unique models across waves: W1-5: p<0.001, W1-2: p=0.010, W2-3: p=0.955, W3-4: p=0.178, W4-5: p=0.516.

b Wilcoxon signed rank test p-values for the prevalence of unique products across waves: W1-5: p<0.001, W1-2: p=0.016, W2-3: p=0.580, W3-4: p=0.488, W4-5: p=0.327.

**Table 2:** Characteristics of unique device models and liquid products identified across five waves of the Vaping and Patterns of E-cigarette Use Research study, an online longitudinal survey of adults who frequently use ENDS

	*W1*	*W2*	*W3*	*W4*	*W5*
*Unique device models*	*N=279 n (%)*	*N=320 n (%)*	*N=319 n (%)*	*N=345 n (%)*	*N=357 n (%)*
**Device types**					
Disposable device	46 (16.5)	81 (25.3)	112 (35.1)	128 (37.1)	129 (36.1)
Reusable device with disposable pod	18 (6.5)	14 (4.4)	9 (2.8)	11 (3.2)	11 (3.1)
Reusable device with refillable pod	65 (23.3)	84 (26.3)	89 (27.9)	88 (25.5)	107 (30.0)
Refillable tank device	150 (53.8)	141 (44.1)	109 (34.2)	118 (34.2)	110 (30.8)
***Unique liquid products***	***N=546 n (%)***	***N=620 n (%)***	***N=638 n (%)***	***N=661 n (%)***	***N=695 n (%)***
**Liquid container type**					
Disposable device	71 (13.0)	136 (21.9)	183 (28.7)	239 (36.2)	298 (42.9)
Disposable pod	31 (5.7)	30 (4.8)	22 (3.5)	26 (3.9)	19 (2.7)
Liquid bottle	444 (81.3)	454 (73.2)	433 (67.9)	395 (59.9)	378 (54.4)
**Flavor**					
Tobacco	45 (8.2)	42 (6.8)	37 (5.8)	21 (3.2)	31 (4.5)
Menthol/mint	38 (7.0)	46 (7.4)	51 (8.0)	59 (8.9)	58 (8.4)
Sweet	443 (81.1)	508 (81.9)	511 (80.1)	554 (83.8)	570 (82.0)
Other	19 (3.5)	22 (3.6)	25 (3.9)	19 (2.9)	20 (2.9)
Missing	1 (0.2)	2 (0.3)	14 (2.2)	8 (1.2)	16 (2.3)
**Nicotine concentration,** median (range)	12 (1.5–60)	18 (1.5–60)	24 (0–60)	35 (3–60)	50 (3–60)
**Formulation**					
Freebase	321 (58.8)	312 (50.3)	302 (47.3)	260 (39.3)	237 (34.1)
Salt	225 (41.2)	308 (49.7)	336 (52.7)	401 (60.7)	458 (65.9)
**PG/VG**					
PG ≤30%/VG ≥70%	349 (63.9)	424 (68.4)	477 (74.8)	282 (42.7)	237 (34.1)
PG >30% and <50%/VG >50% and <70%	49 (9.0)	38 (6.1)	38 (6.0)	248 (37.5)	25 (3.6)
PG ≥50% /VG ≤50%	148 (27.1)	158 (25.5)	123 (19.3)	131 (19.8)	433 (62.3)

In W5, the top five most used ENDS device brands were Elf bar, Smok, Geekvape, Vaporesso, and Vuse; this is a shift from W1 in which the top device brands were Smok, JUUL, Geekvape, Vuse, and Voopoo (Table 3). The top six most used device models in W5 were Elf bar BC5000 (n=160), Vuse Alto (n=74), JUUL (n=60), Geekvape Aegis L200 (n=29), Geekvape Aegis Legend (n=26), and Lost Mary OS5000 (n=26). In W1, these were JUUL (n=160), Vuse Alto (n=78), Geekvape Aegis Legend (n=48), Smok Novo 2 (n=41), and Voopoo Drag 2 (n=36). While 38 (3.2%) of the 1179 participants in W1 used a device that would later be authorized (NJOY Ace, NJOY Daily, Vuse Vibe, Vuse Ciro, and Logic Power), only 17 (1.3%) of the 1290 participants in the W5 sample used one of the authorized devices (NJOY Ace and Vuse Vibe).

**Table 3:** Top five device and liquid brands across five waves of the Vaping and Patterns of E-cigarette Use Research study, an online longitudinal survey of adults who frequently use ENDS

	*W1 (N=1179)*		*W2 (N=1187)*		*W3 (N=1219)*		*W4 (N=1224)*		*W5 (N=1290)*	
	*Brand*	*n (%)*	*Brand*	*n (%)*	*Brand*	*n (%)*	*Brand*	*n (%)*	*Brand*	*n (%)*
**Device brands**	Smok	249 (21.1)	Smok	242 (20.4)	Smok	229 (18.8)	Smok	191 (15.6)	Elf bar	192 (14.9)
	JUUL	160 (13.6)	Geekvape	148 (12.5)	Geekvape	139 (11.4)	Geekvape	152 (12.4)	Smok	181 (14.0)
	Geekvape	136 (11.5)	JUUL	118 (9.9)	Vuse	124 (10.2)	Vuse	96 (7.8)	Geekvape	148 (11.5)
	Vuse	87 (7.4)	Vuse	110 (9.3)	JUUL	98 (8.0)	Voopoo	84 (6.9)	Vaporesso	81 (6.3)
	Voopoo	65 (5.5)	Voopoo	73 (6.1)	Voopoo	79 (6.5)	Elf bar	82 (6.7)	Vuse	76 (5.9)
**Liquid brands**	JUUL	133 (11.3)	JUUL	105 (8.8)	Vuse	117 (9.6)	Vuse	88 (7.2)	Elf bar	192 (14.9)
	Vuse	85 (7.2)	Vuse	101 (8.5)	JUUL	90 (7.4)	Elf bar	81 (6.6)	Vuse	65 (5.0)
	Naked 100	60 (5.1)	Naked 100	42 (3.5)	Hyde	61 (5.0)	JUUL	68 (5.6)	JUUL	47 (3.6)
	Puff bar	42 (3.6)	Puff bar	26 (2.2)	Juice Head	27 (2.2)	Hyde	40 (3.3)	Lost Mary	35 (2.7)
	NJOY	31 (2.6)	NJOY	24 (2.0)	Monster vape labs	24 (2.0)	Monster vape labs	38 (3.1)	Juice Head	31 (2.4)

### ENDS liquids

The number of unique liquid products identified increased across waves from 546 in W1 to 695 in W5 (p<0.001); however, the number of liquid brands decreased from 243 in W1 to 206 in W5. The average number of liquid products per brand increased across waves (Table 1).

Of note, the percent of participants’ liquid products that could be identified increased across waves: 62.8% (n=740) of participants in W1; 69.2% (n=821) in W2; 71.2% (n=868) in W3; 70.8% (n=867) in W4; and 77.4% (n=998) in W5. As a result, the percent of identified products that were unique decreased slightly from W1 to W5; W1: 546/740 (73.8%), W2: 620/821 (75.5%), W3: 638/868 (73.5%), W4: 661/867 (76.2%), W5: 695/998 (69.6%). Additionally, the number of participants across waves increased (W1: n=1179; W5: n=1290); when we standardize the number of unique products by dividing by the sample size (number of products/n), the number of unique liquid products identified per person increased (W1: 546/1179=0.46; W5: 695/1290=0.54).

Across all waves, most unique liquid products were sweet flavored (80.1–83.8%). The median nicotine concentration of the unique liquid products increased across waves, from 12 mg/mL in W1 to 50 mg/mL in W5. The percentage of liquids using salt nicotine increased from 41.2% in W1 to 65.9% in W5. Additionally, the number of liquids with PG ≥50% (VG ≤50%) increased, while the number of liquids with PG ≤30 (VG ≥70) decreased (Table 2). In W5, the top five liquid brands used were Elf bar, Vuse, JUUL, Lost Mary, and Juice Head; in W1, these were JUUL, Vuse, Naked 100, Puff bar, and NJOY (Table 3). The five most common liquid products used in W5 were: Vuse Alto 5%; Menthol pods (salt 50/50 PG/VG, n=29); JUUL 5% Menthol pods (salt 30/70 PG/VG, n=12); Elf bar 5% Blue Razz Ice device (salt 50/50 PG/VG, n=11); JUUL 5% Virginia Tobacco pods (salt 30/70 PG/VG, n=11); and Vuse Alto 5% Golden Tobacco pods (salt 50/50 PG/VG, n=11). In W1, the most common liquid products were JUUL 5% Menthol pods (salt 30/70 PG/VG, n=38); Vuse Alto 5% Menthol pods (salt 50/50 PG/VG, n=26); JUUL 5% Virginia Tobacco pods (salt 30/70 PG/VG, n=15); Vuse Alto 5% Golden Tobacco pods (salt 50/50 PG/VG, n=15); and NJOY Ace 5% Menthol pod (salt 50/50 PG/VG, n=9). Three (0.3%) participants in W1 used a liquid that would later be authorized (Vuse Ciro Original 1.5% and Vuse Vibe Original 3%). Six (0.5%) participants in the W5 sample used one of the authorized liquids (NJOY Ace Classic Tobacco 5% and Vuse Vibe Original 3%).

## DISCUSSION

Despite FDA’s substantial progress in reviewing and providing decisions on PMTA applications, the proportion of participants using devices that were authorized as of W5 decreased across waves, from 3.2% to 1.3%; the proportion of participants using one of the authorized liquids increased slightly following the MGO determinations, from 0.3% to 0.5%. The fact that the proportion of participants using devices that were eventually authorized did not increase following the authorizations seems to suggest that, as of 2023, whether or not a device is authorized is not a substantial factor in determining which devices participants use. While just 1.3% and 0.5% of the 1290 W5 participants used one of the devices or liquids issued an MGO^14^, respectively, the W5 sample included over 190 participants using Elf Bar products (has been issued a warning letter from FDA)^15^, 181 using Smok products, 148 using Geekvape products, 74 using Vuse Alto, and 60 using JUUL (issued a marketing denial order which is being challenged in court)^16^. Some W5 participants, particularly those using tank devices, maybe using devices or liquids purchased prior to denial orders. It is also possible that some or all Smok and Geekvape products are ‘deemed products’, meaning they were on the market on or prior to 8 August 2016, submitted PMTAs by 9 September 2020, and FDA has not yet issued a determination on the product; these products may not currently be subject to enforcement action, pending determinations by FDA. An FDA determination made after data collection for this study denied several menthol- and berry-flavored Vuse Alto flavors^17^; however, the regulatory status of other Vuse Alto products (e.g. tobacco-flavored) remains unclear. Of note, products for which FDA did not file, did not accept, or issued a denial order cannot legally be sold. Public determinations on these brands from FDA would aid in understanding compliance and needed enforcement actions. Further, a study analyzing FDA warning letters to ENDS companies found that a majority (97.4%) were sent to small online retailers^18^; additional enforcement actions targeting larger, more widely used brands may be needed to effectively regulate ENDS according to FDA determinations. Additional research should inform enforcement efforts by examining the devices and liquids that should be prioritized (e.g. products that are most commonly used, most harmful, or most appealing to youth) and the sources through which individuals obtain unauthorized products.

The number of unique ENDS device models and liquid products used by people frequently using ENDS continues to increase. From 2020 to 2023, data from this study show a 28% increase in the number of unique device models and a 27.3% increase in the number of unique liquid products used among our sample. Accordingly, the number of device brands used by participants increased. These findings are supported by other research showing an increase in the number of ENDS products and brands sold and in the proportion of ENDS sold containing at least 5% nicotine^8-10^. Another study examining ENDS brand websites reported a decrease in the number of brands identified from 466 in 2013 to 433 in 2017; however, this study occurred much earlier, prior to the start of PMTA reviews^19^. Further consideration is warranted to understand why the number of devices and liquids used by participants is so high, reaching 357 unique device models and 695 unique liquid products in W5 (Feb–Apr 2023), even though only 23 ENDS products have been authorized for sale in the US.

The percent of unique device models that were disposable increased substantially (17% to 36%) while the percent of refillable tank devices decreased (54% to 31%, respectively). This is in line with other literature showing rising sales and use of disposable devices in the US^8,20-22^. Additionally, the median nicotine concentration of the liquids in our sample increased substantially between waves 1 and 5, from 12 to 50 mg/mL. In line with the rising nicotine concentration, the proportion of liquids using salt nicotine increased from 41% to 66% and the proportion of liquids containing ≥50% PG (≤50% VG) increased from 27% to 62%. It is likely these three shifts are related given that disposable devices tend to have higher nicotine concentrations and to use nicotine salts (which help to reduce the harshness of the nicotine)^13,23-25^, and given the relationship between nicotine concentration and PG/VG ratio^26^. A combination of factors may have contributed to this proliferation in disposable ENDS models. Namely, FDA’s priority enforcement of flavored disposable pod devices may have encouraged those using ENDS to switch to disposable devices, which were more readily available in non-tobacco flavors^27^. Additionally, news sources suggest disposable ENDS companies may be capitalizing on the ease of renaming and/or launching new disposable ENDS brands and models in order to bypass enforcement actions^28,29^. Further consideration is needed on how to effectively enforce current ENDS regulations, particularly for disposable devices and with consideration of resource constraints and the urgency of addressing uptake of ENDS in nicotinenaïve individuals.

Though the number of liquid products increased, the number of liquid brands decreased; the corresponding increase in the number of liquid products per brand may suggest rising popularity of certain brands and expanded use of various products within those brands. Among the unique liquids in our sample, sweet was the most common flavor across all waves (80–84%); this aligns with current literature showing the high popularity of sweet flavors among ENDS users^30-33^. FDA may consider prioritizing enforcement against sweet-flavored products given their popularity and importance in youth use^30-35^.

### Strengths and limitations

Each wave of the VAPER study involved >1100 adults who frequently use ENDS; in total, data from 2987 individuals are included here. The substantial sample size of this study is one strength of these analyses. Additionally, the VAPER sample closely resembles the national sample of adults who use ENDS daily, captured by the 2019 TUS-CPS^11^. The data reported here were captured directly from participant photos of their devices and liquids and from manufacturer, academic, retail, and review sites; therefore, these data are only as accurate as the photos and sites utilized for coding. However, previous research has suggested that self-reported and photo-coded device type and flavor have a high agreement, suggesting a fair level of accuracy with these data^36^. As mentioned in the results, the increased identification of products across waves may account for some of the increase in liquid products across waves; however, the increase in the proportion of liquids identified is more likely driven by the decreasing prevalence of disposable pod devices, for which photos often do not include the flavor or nicotine concentration of the product because this information is often not included on the pod itself. This is supported by the fact that JUUL accounted for 67 of the unidentified products in W1 but just 16 in W5; Vuse accounted for 18 unidentified in W1 and 8 in W5; and NJOY accounted for 20 unidentified in W1 and 5 in W5; combined, about half of the difference (76 of 147) in unidentified liquids between W1 and W5 is accounted for by these three brands alone, each of which has few product options. While the increasing number of participants across waves could also account for some of the increase in the number of device models and liquid products identified, when we standardize the number of unique models or products by dividing by the sample size (number of models or products/n), the number of unique device models and products identified per person also increased, indicating the increasing sample size is not the only factor in the increasing number of unique device models or liquid products across waves. Additionally, given this study focused on changes in the number of unique devices and liquids across waves and the implications for regulatory and enforcement efforts, results on the changes in the characteristics of unique devices and liquids are meant to be descriptive; formal statistical comparisons were not conducted for these results (Table 2).

## CONCLUSIONS

The findings of this study highlight the importance of continued monitoring and enforcement of ENDS in the US, particularly as the FDA has reviewed the vast majority of PMTAs submitted. Only 23 ENDS products have been authorized for sale by the FDA, yet as recently as Feb–Apr 2023, 357 unique device models and 695 unique liquid products were identified among a sample of adults who frequently use ENDS. In fact, from 2020 to 2023, the number of unique ENDS devices and liquids used by VAPER study participants increased. Additionally, the percentage of unique devices that were disposable and liquids that used nicotine salts increased, as did the median nicotine concentration of the liquids. Further research and monitoring are needed to inform how FDA prioritizes enforcement actions and what types of enforcement actions are effective. Additionally, sweet flavors may be considered an enforcement priority, given their popularity and relevance in youth ENDS use.

## Data Availability

The data supporting this research are available from the authors on reasonable request.
